# NBAT1 suppresses breast cancer metastasis by regulating DKK1 via PRC2

**DOI:** 10.18632/oncotarget.5609

**Published:** 2015-09-10

**Authors:** Pengnan Hu, Junjun Chu, Yanqing Wu, Lijuan Sun, Xiaobin Lv, Yinghua Zhu, Jingjing Li, Qiannan Guo, Chang Gong, Bodu Liu, Shicheng Su

**Affiliations:** ^1^ Guangdong Provincial Key Laboratory of Malignant Tumor Epigenetics and Gene Regulation, Sun Yat-sen Memorial Hospital, Sun Yat-sen University, Guangzhou, China; ^2^ Key Laboratory of Gene Engineering of Ministry of Education, State Key Laboratory of Biocontrol, School of Life Sciences, Sun Yat-sen University, China; ^3^ Breast Tumor Center, Sun Yat-sen Memorial Hospital, Sun Yat-sen University, Guangzhou, China

**Keywords:** LncRNA, NBAT1, breast cancer, metastasis

## Abstract

Long noncoding RNA NBAT1 (neuroblastoma associated transcript 1) regulates cell proliferation and invasion by interacting with PRC2 (polycomb repressive complex 2) member EZH2 (enhancer of zeste 2). Decreased expression of NBAT1 is associated with poor clinical outcome in neuroblastomas. However, the roles of NBAT1 in other cancers remain unknown. Here, we report that NBAT1 is down-regulated in various types of cancer. Particularly, reduced NBAT1 in breast cancer is associated with tumor metastasis and poor patient prognosis. In vitro, ectopic NBAT1 inhibits migration and invasion of breast cancer cells. Mechanistic study shows that NBAT1 is associated with PRC2 member EZH2 and regulates global gene expression profile. Among them, DKK1 (dickkopf WNT signaling pathway inhibitor 1) is found to be regulated by NBAT1 in a PRC2 dependent manner, and is responsible for NBAT1's effects in inhibiting migration and invasion of breast cancer cells. Taken together, our study demonstrates that long noncoding RNA NBAT1 is a potential breast cancer prognostic marker, as well as a potential therapeutic target to inhibit breast cancer metastasis.

## INTRODUCTION

Long non-coding RNAs (lncRNAs) have emerged as new regulators for cancer biology by participating in both oncogenic and tumor suppressing pathways. Previous studies have demonstrated the potential involvement of lncRNAs in the formation and progression of multiple types of cancer, including breast cancer [1]. For example, oncofetal H19 RNA [2] and TreRNA promote breast cancer metastasis by inducing Epithelial-Mesenchymal Transition (EMT) of cancer cells [3]. Conversely, NKILA (NF-κB Interacting LncRNA) inhibits breast cancer progression and metastasis by suppressing NF-κB activation [4]. Therefore, lncRNAs may play critical roles in breast cancer biology, including metastasis.

EZH2 is a major component of Polycomb Repressive Complex 2 (PRC2), which is regulated by several lncRNAs, such as Xist (X inactive specific transcript) [5], and HOTAIR (HOX transcript antisense RNA) [6]. Tissue microarray analysis demonstrated that EZH2 protein levels were strongly associated with aggressiveness of breast cancer. On the other hand, over-expression of EZH2 in immortalized human mammary epithelial cell lines promotes anchorage independent growth and cell invasion [7]. Furthermore, there are evident associations between EZH2 and markers of tumor cell proliferation, as well as the phenotypes of aggressive diseases [8]. In addition, new findings have demonstrated that aberrant up-regulation of EZH2 expression in neuroblastoma cells lead to silencing of several tumor suppressors, which contributed to the oncogenesis and maintenance of the undifferentiated phenotype of neuroblastoma tumors [9].

NBAT1 is identified in neuroblastoma as a tumor-suppressing lncRNA. Loss of NBAT1 contributes to the aggressiveness of neuroblastoma by promoting proliferation and by impairment of differentiation of neuronal precursors [10]. Furthermore, interaction of NBAT1/EZH2 is indespensible for NBAT1's function in suppressing expressions of its target genes, which are implicated in cell proliferation and cell migration [11]. However, NBAT1's roles in other cancers remain unknown. Therefore, in the present study, we explored the functions and mechanisms of NBAT1 in breast cancer.

## RESULTS

### NABT1 reduction predicts poor clinical outcomes in breast cancer patients

**Figure 1:**
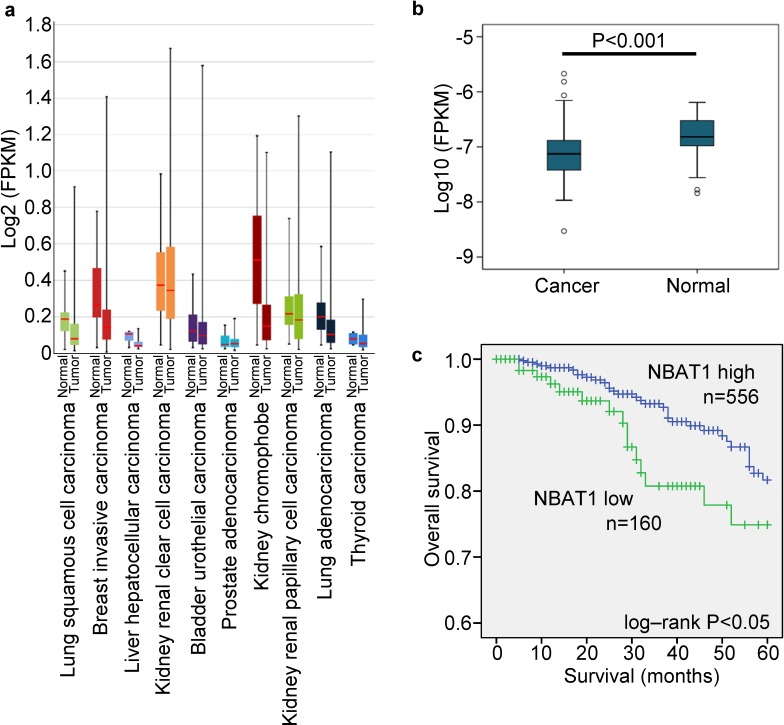
NBAT1 reduction predicts poor clinical outcome in breast cancer patients **a**. The expression of NBAT1 in different tumor. **b**. The expression of NBAT1 in breast cancer and normal breast tissue. **c**. Kaplan-Meier survival curve of patients with breast cancer with low and high NBAT1.

By analyzing the expression of NBAT1 in the lncRNA expression database lncRNAtor, which is based on The Cancer Genome Atlas (TCGA) [12], we found that NBAT1 expression was down-regulated in the cancerous tissues compared to their normal counterparts. This was observed in many types of cancers including invasive breast cancer, lung squamous cell carcinoma, hepatic cell carcinoma, chromophobe renal cell carcinoma, and lung adenocarcinoma (Figure 1a). We then focused on breast carcinoma for in-depth investigations. In an analysis based on multiple data sets collected from a previous study [12], NBAT1 expression was significantly lower in cancerous breast tissues than in normal ones (*p* < 0.001). Additionally, NBAT1 was negatively correlated with ER (*p* < 0.001) and PR (*p* < 0.001) expressions, and was also lower in patients who had received combined treatments of Hormone Replacement Therapy (HRT) and chemotherapy than chemotherapy alone (Table 1). To further validate these findings, we have analyzed data retrieved directly from TCGA involving 814 patients. In accordance to the previous findings, the expressions of NBAT1 in breast cancers were significantly lower than those in normal breast tissues (*p* < 0.001) (Figure 1b). Reduced NBAT1 was also significantly correlated with lymph node metastasis, as well as post-menopause status (Table 2). More importantly, Kaplan-meier survival analysis based on NBAT1 expression showed that low NBAT1 expression was correlated with poorer patient survival (Figure 1c). Taken together, these data indicated that down-regulation of NBAT1 expressions in breast cancer tissues was correlated with metastasis and poor patient prognosis.

**Table 1:** Dataset of NBAT1 Differential Expression

Dataset Title	Sample Count	Data Source	P-value	Compare (A vs B)	FoldChange (A over B)
Breast invasive carcinoma: Breast carcinoma estrogen receptor status	754	TCGA	2.60E-08	Negative vs Positive	1.38
Breast invasive carcinoma: Combination of Chemotherapy and Hormone therapy	328	TCGA	3.62E-02	Chemotherapy vs Hormone therapy,chemotherapy	1.36
Breast invasive carcinoma: Drug Arimidex or not	325	TCGA	6.53E-02	Arimidex vs No_Arimidex	0.8
Breast invasive carcinoma: Normal vs Tumor	894	TCGA	9.03E-14	Normal vs Tumor	2.01
Breast invasive carcinoma: Progesterone receptor status	751	TCGA	5.70E-06	Negative vs Positive	1.27
Uterine corpus endometrioid carcinoma: Histological type	301	TCGA	4.72E-04	Endometrioid endometrial adenocarcinoma vs Serous endometrial adenocarcinoma	0.47
Uterine corpus endometrioid carcinoma: Person neoplasm cancer status	283	TCGA	2.72E-02	Tumor free vs With tumor	0.37

**Table 2:** Correlation of NBAT1 Expressing with Clinicopathological Status in 716 Cases of Patients with Breast Cancer

clinicopathological status	NBAT1		χ^2^	P value
	high	low		
T				
1	156	38	3.39	0.335
2	326	94		
3	53	19		
4	18	9		
Indeterminate^2^	2	1		
N				
0	269	63	7.09	0.069
1	187	55		
2	63	27		
3	26	12		
Indeterminate^2^	10	4		
M				
0	511	140	1.20	0.272
1	10	5		
Indeterminate^2^	35	15		
stage				
1	96	22	6.70	0.082
2	324	85		
3	111	45		
4	9	5		
Indeterminate^2^	14	5		
ER				
(−)	124	34	0.04	0.847
(+)	409	117		
Indeterminate^2^	24	8		
PR				
(−)	175	46	0.35	0.554
(+)	355	105		
Indeterminate^2^	21	14		
HER2				
(−)	302	74	1.41	0.236
(+)	88	29		
Indeterminate^2^	168	55		
LN metastasis				
(−)	269	63	4.17	0.041
(+)	276	94		
Indeterminate^2^	10	4		
menopause status^1^				
Peri	23	5	14.49	0.002
Post	330	121		
Pre	140	25		
Indeterminate^2^	63	9		

1 menopause status, Indeterminate: neither Pre or Postmenopausal, Peri: 6-12 months since last menstrual period, Post: prior bilateral ovariectomy OR >12 mo since LMP with no prior hysterectomy, Pre: <6 months since LMP AND no prior bilateral ovariectomy AND not on estrogen replacement.

2 Statistics do not include Indeterminate group)

### NBAT1 inhibits migration and invasion of breast cancer cells

**Figure 2:**
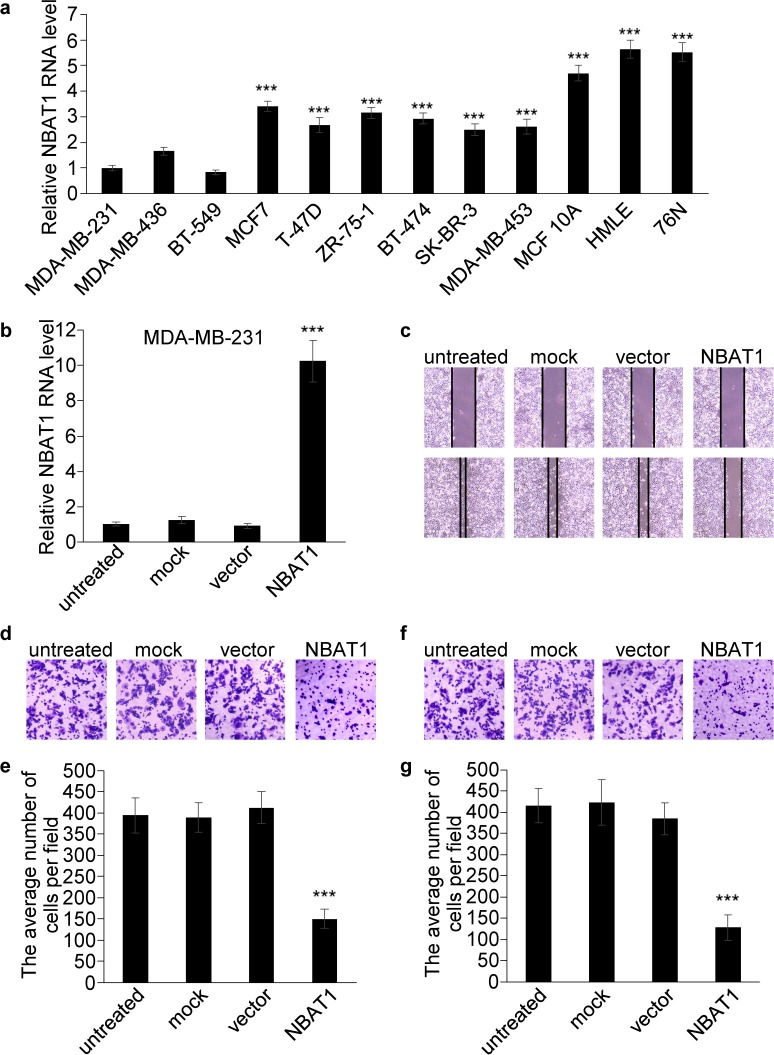
Over-expression NBAT1 inhibits the migration and invasion of MDA-MB-231 **a**. The expression levels of NBAT1 were determined by using real-time PCR in breast cancer cell lines with high (MDA-MB-231, MDA-MB-436 and BT-549) and low (MCF7,T-47D,ZR-75-1, BT-474, SK-BR-3 and MDA-MB-453) invasion potential, and in breast epithelial lines (MCF 10A, HMLE and 76N). **b.** The expression levels of NBAT1 were determined in MDA-MB-231 with over-expression NBAT1. (mean±SD, n=3, *** p < 0.001 versus MDA-MB-231) **c**. MDA-MB-231 transfection of NBAT1 decreased directional migration compared to empty vector transfected, mock or untreated cells (controls) in wound healing assays. **d.**, **f.**, Representative images are shown of a Boyden chamber assay for migrated (d) and invaded (f) MDA-MB-231 cells transfected with untreated, mock, vector or NBAT1. **e.**, **g**., Histogram showing that the number of migrated (e) and invaded (g) cells transfected with NBAT1 was significantly lower than for control groups (untreated, mock and vector, mean±SD, n=3, *** P < 0.001).

To further investigate the roles of NBAT1 in breast cancer, we examined the expressions of NBAT1 in immortalized non-tumorigenic human mammary epithelial cell lines and breast cancer cell lines with different invasive potentials. Compared with mammary epithelial cell lines, NBAT1 expression was down-regulated in the breast cancer cells (Figure 2a). Furthermore, expression of NBAT1 was negatively associated with invasive potential of breast cancer cell lines. The cells with higher migration potentials (MDA-MB-231, MDA-MB-436 and BT-549) expressed significantly lower NBAT1 compared to cells with lower migration potentials (MCF7, T-470, ZR-75-1, BT-474, SK-BR-3 and MDA-MB-453) (Figure 2a).

**Figure 3:**
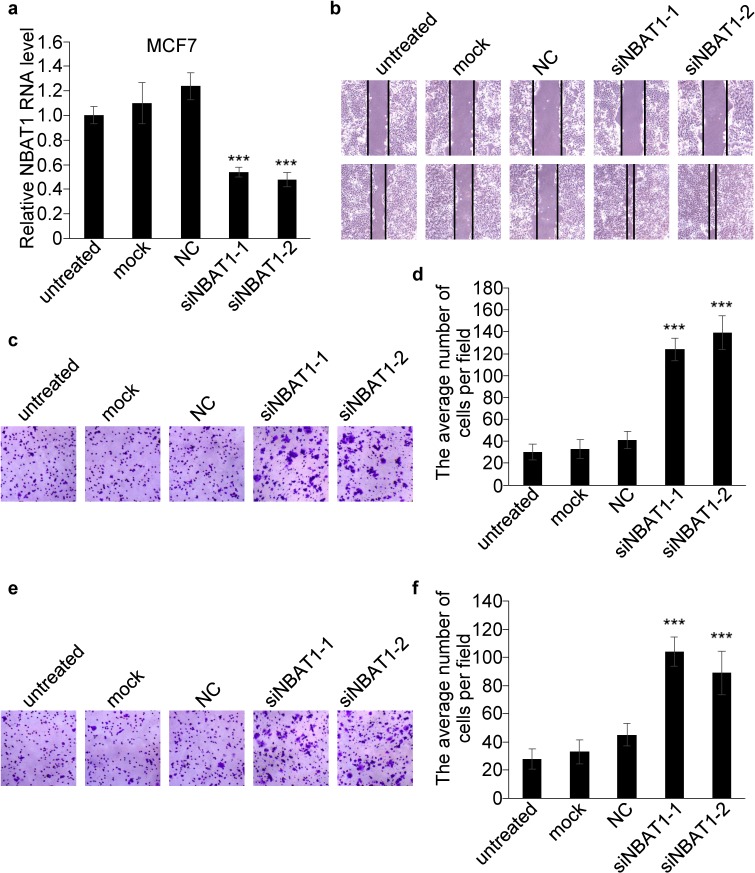
Silencing NBAT1 promotes migration and invasion of MCF7 cells **a**. The expression levels of NBAT1 were determined in MCF7 with knock down NBAT1. **b**. Knock-down NBAT1 increased directional migration compared to NC, mock or untreated cells (controls) in wound healing assays. **c.**, e., Representative images are shown of a Boyden chamber assay for migrated (c) and invaded (e) MCF7 cells transfected with untreated, mock or siRNA-NC or siNBAT1. **d.**, **f.**, Histogram showing that the number of migrated (d) and invaded (f) cells with knock-down NBAT1 was significantly higher than for control groups (untreated, mock and vector, mean±SD, n=3, *** P < 0.001).

The correlation of NBAT1 expression with invasiveness suggests NBAT1 might be involved in the regulation of invasiveness of breast cancer cells. We further investigated whether NBAT1 mediates breast cancer cell migration and invasion using Boyden chamber assay with or without Matrigel coating, respectively, as well as the wound healing assay. We ectopically expressed NBAT1 in highly invasive MDA-MB-231 cells with pcDNA 3.1 vector (Figure 2b). Wound healing and transwell assays suggested that exogenous NBAT1 suppressed both migration and invasion of MDA-MB-231 cells (Figure 2c-2g). On other hand, silencing NBAT1 by siRNAs in MCF7 cells significantly reduced NBAT1 expression levels (Figure 3a), which was accompanied by significantly increased migration and invasion of MCF7 cells (Figure 3b-3f). These data suggest that NBAT1 represses breast cancer cell migration and invasion.

### Over-expression of NBAT1 in MDA-MB-231 cells results in global gene expression profile change

**Figure 4:**
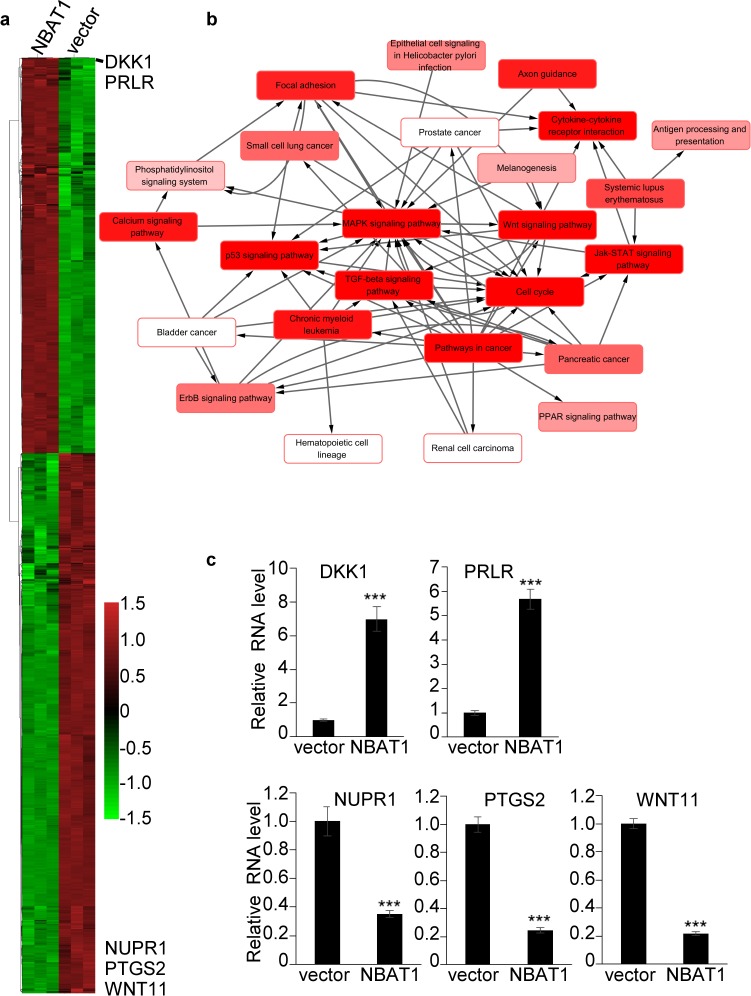
Over-expression of NBAT1 in MDA-MB-231 cells results in global gene expression profile change **a**. Heatmap representing hierarchical clustering of all dysregulated genes whose relative fold changes are more than 2 times compared MDA-MB-231/NBAT1 with MDA-MB-231/vector cells. **b**. Pathway-network analysis of the significant pathways of the differential expression genes. (Lines represent the relationship between the pathways, red to white represents the P value; the smaller the P value is, the deeper the red is.) **c**. The expression levels of DKK1, PRLR, NUPR1, PTGS2, WNT11 were determined in MDA-MB-231 with over-expression NBAT1 by qRT-PCR (mean±SD, n=3, *** p < 0.001, versus vector).

To further investigate the mechanisms of how NBAT1 represses migration and invasion of breast cancer cells, we explored global gene expression changes affected by forced expression of NBAT1 in MDA-MB-231 cells by microarrays. The results showed a significant global change in gene expression caused by over-expression of NBAT1, with 1594 genes down-regulated and 975 up-regulated at a cut-off of 2.0 folds (Figure 4a). To further identify the cellular functions which are regulated by NBAT1, we conducted a pathway-network analysis. The results suggested that forced expression of NBAT1 affected WNT signaling pathway, TGF-beta signaling pathway, and MAPK signaling pathway, etc. (Figure 4b). As was previously reported, NBAT1 regulates gene expression by modulating the functions of PRC2. Among them, we further checked the expression levels of DKK1, PRLR, NUPR1, PTGS2, WNT11 by qRT-PCR and the results were consistent with the array (Figure 4c). Moreover, DKK1, an inhibitor of WNT signaling pathway, was found to be the second highest fold-changed gene.

### NBAT1 inhibits migration and invasion of breast cancer cells by activating DKK1 expression

**Figure 5:**
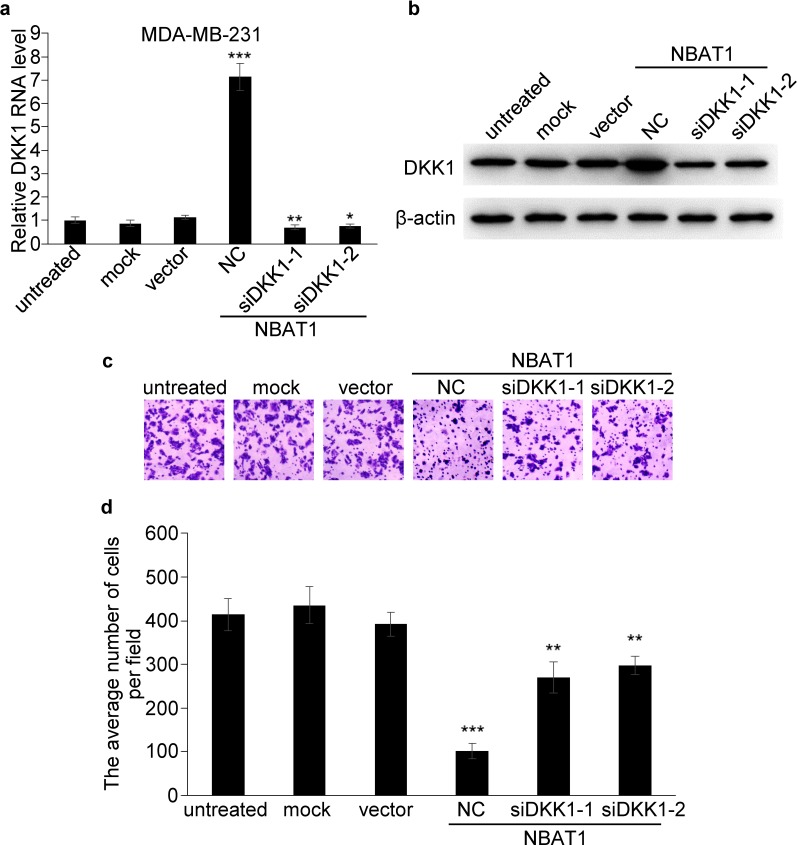
NBAT1 inhibits invasion of breast cancer cells by activating DKK1 expression **a.**, **b**. qRT-PCR and western blot analysis for DKK1 in NBAT1-expression MDA-MB-231 cells transfected with siRNA targeting DKK1 (NC, siDKK1-1 and siDKK1-2). **c**. Representative images of Boyden chamber assay for invaded cells (over-expression NBAT1 while inhibit DKK1). **d**. Histogram showing that the number of invaded cells with knockdown DKK1 was significantly higher than for NC, and similar to control groups (untreated, mock and vector, mean±SD, n=3, * P < 0.05, ** P < 0.01, *** P < 0.001).

We then examined whether DKK1 played an important role in NBAT1 mediated suppression of cell migration and invasion. Transfection of siRNAs targeting DKK1 in MDA-MB-231 cells completely reversed the up-regulation of DKK1 induced by NBAT1 (Figure 5a, 5b). And knock-down of NBAT1-upregulated DKK1 has partially reversed the effects of NBAT1-regulated cellular invasion (Figure 5c, 5d). These data suggest that the repression effects of NBAT1 on cell migration and invasion is mediated, at least in part, through DKK1.

### NBAT1 inhibits migration and invasion of breast cancer cells via EZH2

**Figure 6:**
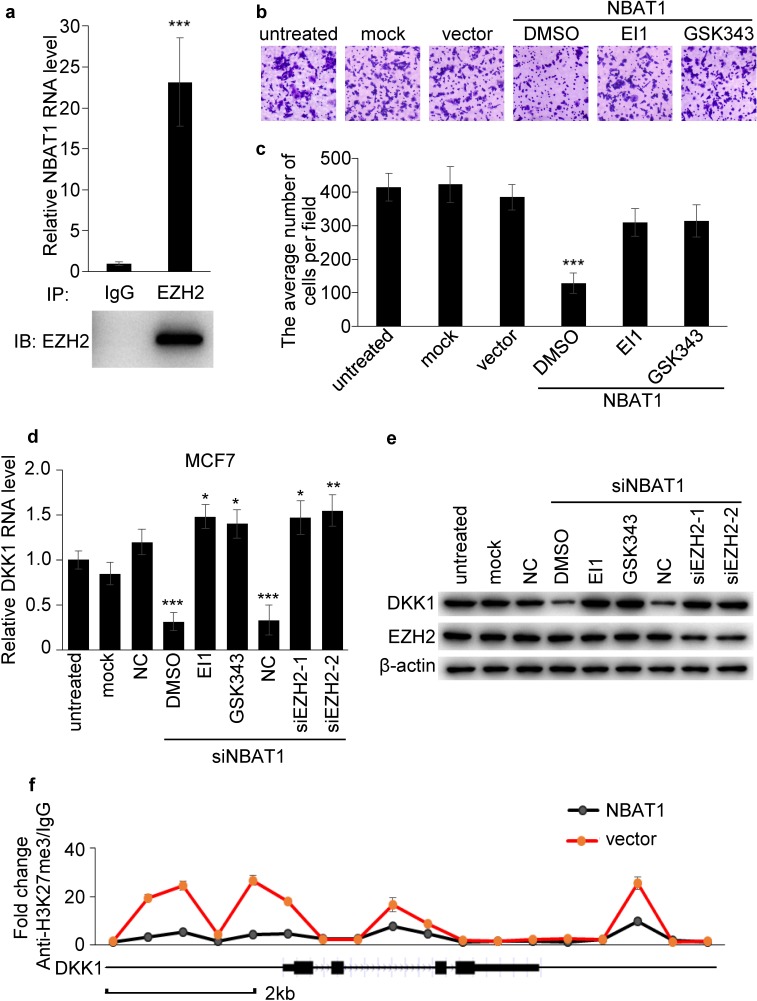
NBAT1 inhibits invasion of breast cancer cells via EZH2 **a**. Binding of NBAT1 to EZH2 complex in MDA-MB-231 cells, shown by RNA immunoprecipitation followed qRT-PCR (mean±SD, n=3, *** p < 0.001 versus lgG). **b**. Representative images of Boyden chamber assay for invaded cells. (Over-expression NBAT1 while inhibit EZH2 with EI1 or GSK343 in MDA-MB-231.) **c**. Histogram showing that the number of invaded cells with inhibited EZH2 was significantly higher than for DMSO, and similar to control groups (untreated, mock and vector, mean±SD, n=3, *** P < 0.001). **d.**, **e**. qRT-PCR and western blot analysis for DKK1 in NBAT1-knockdown MCF7 cells inhibited EZH2 with EI1 or GSK343 or transfected with siRNA targeting EZH2 (NC, siEZH2-1 and siEZH2-2). **f.** ChIP-qPCR analysis for H3K27me3 of *DKK1* gene in NBAT1-expression MDA-MB-231 cells (mean±SD, n=3, *** p < 0.001 versus vector).

Next we sought to determine whether NBAT1 regulates breast cancer cell invasion via EZH2. An RNA immunoprecipitation assay of EZH2 showed that NBAT1 could bind to EZH2 (Figure 6a). In order to test whether NBAT1 regulates EZH2 functions, we then applied EZH2 inhibitors EI1 or GSK343 in MDA-MB-231 breast cancer cells with NBAT1 over-expression. The invasion suppression effects of NBAT1 was reversed by concomitant EZH2 inhibitors (Figure 6b, 6c). In agreement with the previous result, silencing NBAT1 with siRNAs in MCF7 down-regulated DKK1 in both mRNA and protein levels. However, this effect was abrogated by administration of EZH2 inhibitors or siRNAs targeting EZH2 (Figure 6d, 6e). To further investigate how NBAT1 up-regulated DKK1 via EZH2 in breast cancer, we applied chromatin immunoprecipitation (ChIP)-quantitative real-time PCR (ChIP-qPCR) to analyze trimethylation of histone H3 lysine 27 (H3K27me3) status at *DKK1* gene, which is a marker of suppressed chromatin [13-15]. Exogenous over-expression of NBAT1 decreased H3K27me3 level of *DKK1* promoter in MDA-MB-231 cells (Figure 6f). This indicates that NBAT1 suppresses EZH2-induced H3K27me3 of *DKK1*. These data suggest that the effect of NBAT1 on cell invasion and DKK1 expression is mediated through repression of EZH2 functions.

## DISCUSSION

Although it was previously reported that low expression of NBAT1 was significantly correlated with poor overall and event free survival of neuroblastoma patients [10], the expression of NBAT1 in other cancer was still unknown. Our data showed that NBAT1 was down-regulated in invasive breast cancer, lung squamous cell carcinoma and adenocarcinoma, hepatic cell carcinoma, chromophobe renal cell carcinoma than in normal tissues. Especially, NBAT1 reduction was associated with poor patient survival as well as with lymph node metastases in breast cancer.

EZH2 is the catalytic subunit of PRC2 and often over-expressed in breast cancer [16]. EZH2 regulates invasion of breast cancer [7, 8] mainly through mediating transcriptional silencing of the tumor suppressor genes, including E-cadherin by regulating histone modifications in the promoters of these target genes, including H3K27me3 [17]. And the PRC2-targeted protein E-cadherin was significant decreased in lymph node metastases, suggesting PRC2 might contribute to epithelial-mesenchymal transition (EMT) in lymph node metastatic process through repression of E-cadherin [18]. We found that NBAT1 could inhibit the migration and invasion of breast cancer, while the effect of NBAT1 over-expression on invasion was reversed by concomitant EZH2 inhibitors, suggesting that the effect of NBAT1 on cell migration and invasion might be mediated through EZH2.

Several studies have demonstrated that DKK1, an inhibitor of WNT signaling pathway, could inhibit the migration and invasion of breast cancer [19-25]. In addition, DKK1 was regulated directly by PRC2 through H3K27me3 [26]. Over-expression NBAT1 could down-regulate H3K27me3 level of *DKK1* and significantly increase the expression of DKK1, while the effects of NBAT1 over-expression on invasion was partially reversed by concomitant DKK1 knockdown, suggesting that the effect of NBAT1 on cell migration and invasion is mediated, at least in part, through DKK1.

It was shown that EZH2 bound to lncRNA Xist, and the latter was responsible for recruitment of PRC2 to specific genomic regions [5]. The mechanisms have been further elucidated that the PRC2 complex protein EZH2 specifically recognize the conserved A-repeat domain of Xist RNA [5, 27-29]. Another lncRNA HOTAIR, which is a HOXC cluster-derived lincRNA, was also demonstrated to recruit PRC2 to its target genes [30, 31]. HOTAIR promotes metastasis of breast cancer cells and its over-expression was correlated with poor prognosis of breast cancer patients [32, 33]. Moreover, simultaneous up-regulation of HOTAIR and EZH2 might be of clinically significance because a recent study of a breast cancer archive correlates simultaneous high expression of EZH2 and HOTAIR with aggressive tumor growth and metastasis [34]. Like Xist, HOTAIR also interacted with and recruited PRC2 and regulated chromosome occupancy through its interaction with EZH2 [6]. HOTAIR was also reported to regulate E-cadherin by binding to EZH2 and regulating H3K27me3 of E-cadherin promoter [35]. Moreover, it has been shown that increased expression of only EZH2 did not necessarily correlate with increased abundance of H3K27me3 [36]. This might be explained by the hypothesis that EZH2 bound with different lncRNAs might have different roles. This was supported by our observations: we have found that NBAT1 also interact with EZH2, just like XIST and HOTAIR. However, rather than promoting EZH2 or PRC2 functions in repressing target gene expression via chromatin regulation, NBAT1 repressed EZH2 functions. In agreement with these results, NBAT1 behaved as a tumor-suppressor to repress PRC2-enhanced cell invasion and migration. Therefore, the functions of EZH2 in breast cancer might be strictly regulated by lncRNAs.

Our study has demonstrated that NBAT1 expression was down-regulated in multiple cancer types, suggesting that NBAT1 was a potential tumor-suppressor gene. Specifically, we have demonstrated the correlation of NBAT1 expression with breast cancer metastasis, and therefore the potential value of harnessing it as a lncRNA prognostic marker. Mechanistic study has shown that NBAT1 might exhibit this tumor-suppressing function by modulating the global gene expression regulator PRC2 complex. This point to a possibility of developing RNA-based targeted drugs to interfere with these gene expression regulators to overcome diseases like breast cancer, which calls for in-depth mechanistic studies in the future.

## MATERIALS AND METHODS

### Cell cultures and treatment

MCF-7, T47D, ZR75-1, BT-474, MDA-MB-453, BT-549, SK-BR-3, MDA-MB-231 and MDA-MB-436 breast cancer cell lines as well as MCF-10A immortalized mammary epithelial cell line were obtained from American Type Culture Collection and cultured according to the recommended protocols. HMLE cells were kindly provided by Dr. R.A. Weinberg (Whitehead Institute, Cambridge, MA) and cultured as recommended [37]. 76N cell line was originally supplied and cultured as described by Band et al. [38] For siRNA and plasmid DNA transfection, cells were transfected with specific siRNA duplexes targeting NBAT1 or DKK1 or EZH2 or pcDNA3.1 vector expressing NBAT1 construct using Lipofectamine 2000 (Invitrogen) according to the manufacturer's instruction. To inhibit EZH2 activities, cells were treated with 10μM EI1 (S7611, Selleck) or GSK343 (S7164, Selleck) for 36 h. Specific siRNA sequences are listed in Table 3.

**Table 3:** siRNA sequences

name	sequence
NC-f	5′-UUCUCCGAACGUGUCACGUTT-3′
NC-r	5′-ACGUGACACGUUCGGAGAATT-3′
siNBAT1-1-f	5′-GUCCAAAGUUGUGUUUACUTT-3′
siNBAT1-1-r	5′-AGUAAACACAACUUUGGACTT-3′
siNBAT1-2-f	5′-GUCCAAAGUUGUGUUUACUTT-3′
siNBAT1-2-r	5′-UUGUCUUUCAACCAUGGGCTT-3′
siDKK1-1-f	5′-AAUGGUCUGGUACUUAUUCCGC-3′
siDKK1-1-r	5′-GGAAUAAGUACCAGACCAUUGC-3′
siDKK1-2-f	5′-GGAAUAAGUACCAGACCAUTT-3′
siDKK1-2-r	5′-AUGGUCUGGUACUUAUUCCTT-3′
siEZH2-1-f	5′-CCAUGUUUACAACUAUCAATT-3′
siEZH2-1-r	5′-UUGAUAGUUGUAAACAUGGTT-3′
siEZH2-2-f	5′-GGAUGGUACUUUCAUUGAATT-3′
siEZH2-2-r	5′-UUCAAUGAAAGUACCAUCCTT-3′

### Quantitative real-time PCR

Quantitative real-time PCR (qRT-PCR) was performed by using a Roche LightCycler^®^ 480 Real-Time PCR System. The qRT-PCR amplification protocol was conducted according to the user guide of the SYBR^®^ Green master mixes on the Roche LightCycler^®^ 480 Real-Time PCR System. All samples were tested in triplicate. The data were obtained from 3 independent experiments. The primer sequences are listed in Table 4.

**Table 4:** qRT-PCR primer sequences

name	sequence
NBAT1-f	5′-TCAGCAGAAACGGCACGAT-3′
NBAT1-r	5′-AGATGACCCAGGCACCTCC-3′
DKK1-f	5′-GAGACACTAAACCAGCTATCCAAAT-3′
DKK1-r	5′-ATCACAGGGGAGTTCCATAAAGA-3′
PRLR-f	5′-CCAGCGACCTTCATTCAGATAC-3′
PRLR-r	5′-ACTGCCCAGACAATAATCAAACA-3′
NUPR1-f	5′-AAGGCAGGAGTCAGAGGTGAAGTG-3′
NUPR1-r	5′-GGAAGACAAGCCAGGCACGAT-3′
PTGS2-f	5′-CCGAGGTGTATGTATGAGTGTGG-3′
PTGS2-r	5′-AAATCCCTTGAAGTGGGTAAGTATG-3′
WNT11-f	5′-TGAAGGACTCGGAACTCGTCTATCT-3′
WNT11-r	5′-TACTTACAGTGGCACCGCTCG-3′
β-actin-f	5′-AGCCTCGCCTTTGCCGATCC-3′
β-actin-r	5′-ACATGCCGGAGCCGTTGTCG-3′

### Wound healing assay

Breast cancer cells were cultured under permissive condition to about 90% confluence. A linear wound was created in the confluent monolayer using a pipette tip. Cancer cells were allowed to close the wound for 30 h (MDA-MB-231) or 40 h (MCF7), and observed under microscopy.

### Boyden chamber assay

Invasion and migration of breast cancer cells were examined using 24-well Boyden chambers (BD Biosciences) with 8 μM inserts with or without precoated Matrigel Basement Membrane Matrix (BD Biosciences), respectively. Breast cancer cells (10^5^ cells per well) were placed on the inserts in the upper chambers and cultured at 37°C in 5% CO2. MDA-MB-231 after 7 h (migration assay) or 16 h (invasion assay), MCF-7 after 30 h (migration assay) or 48 h (invasion assay), cells on the upper surface of the membrane filter were removed. The migrated and invaded cells that crossed the inserts to the lower surface were fixed with 4% formaldehyde, stained with crystal violet (0.005%, sigma), and counted as cells per field of view under microscopy.

### Western blotting

Protein extracts were resolved in 10% SDS–polyacrylamide gel electrophoresis, transferred to polyvinylidene difluoride membranes and probed with antibodies against EZH2 (1:500, sc-166609, Santa Cruz), DKK1 (1:500, sc-374574, Santa Cruz) and β-actin (1:1,000, 5125, CST). Peroxidase-conjugated secondary antibody against mouse (1:1,000, 7076, CST) was used, and the antigen–antibody reaction was visualized by the enhanced chemiluminescence assay (WBKLS0500, Millipore).

### RNA immunoprecipitation

RNA immunoprecipitation was performed using Magna RIP RNA-Binding Protein Immunoprecipitation Kit (17-700, Millipore) according to manufacturer's instructions.

### Chromatin immunoprecipitation

Chromatin immunoprecipitation (ChIP) was performed using antibodies against H3K27me3 (9733, CST) and Magnetic Chromatin Immunoprecipitation Kit (53008, Actice Motif) according to manufacturer's instructions. Approximately 1/20 of the immunoprecipitated DNA was used in each qPCR. Gene-specific primers for DKK1 are listed in Table 5. Three replicates for each experimental point were performed. Results were normalized using the internal control IgG.

**Table 5:** ChIP-qPCR primer sequences

name	sequence
primer1-f	5′-GAAAGGGTATTGCGTGGTCCC-3′
primer1-r	5′-CTTAGCGCCTGACTTCCTCAT-3′
primer2-f	5′-CATTTCCCTCTTCCCTCCACC-3′
primer2-r	5′-CAAACGAGGGTAAGAAGGAAGAA-3′
primer3-f	5′-ACTCAGATCTGCAAACTGCGA-3′
primer3-r	5′-GCTGACTGTGCACACAAGTCA-3′
primer4-f	5′-CACCCCTCGGCTCTGTAAAGT-3′
primer4-r	5′-AATAAATGCAGGCGGCAGCAG-3′
primer5-f	5′-CGGGGTGAAGAGTGTCAAAGG-3′
primer5-r	5′-GAGACAACAAAGCCGGGATGG-3′
primer6-f	5′-CCTGCAGTCAGGACTCTGGGA-3′
primer6-r	5′-TGACCGTCACTTTGCAAGCCT-3′
primer7-f	5′-GGAGTGAGCGCCACCTTGAAC-3′
primer7-r	5′-GTACTTATTCCCGCCCGGGTA-3′
primer8-f	5′-ACGAGGGAGTAGAACGTGCTG-3′
primer8-r	5′-GGGCGCTGATCACAGTCCTTA-3′
primer9-f	5′-CAGGCGTGCAAATCTGTCTCG-3′
primer9-r	5′-GGAGCTTTCAGGACTCACCAT-3′
primer10-f	5′-AATGGGTGTTCAGCATGCAGG-3′
primer10-r	5′-ATTGAGGGACACGGAGGAAGA-3′
primer11-f	5′-TGGTACCCTCACAACTTGACT-3′
primer11-r	5′-AGCTGTCTAAATCCACCTTTTGA-3′
primer12-f	5′-CCTACCACAGTTGGTGGGAAA-3′
primer12-r	5′-TGGACAGAAGCACCAATGTGT-3′
primer13-f	5′-TTAGTGAAACGATGCAGGTTT-3′
primer13-r	5′-CCTGAGGCACAGTCTGATGAC-3′
primer14-f	5′-TCTGTGGTTTCAGTTAAGCATTC-3′
primer14-r	5′-AGGTAAGTGCCACACTGAGAA-3′
primer15-f	5′-GGGAAAGCAAACTTCCCAATT-3′
primer15-r	5′-TTGCACGATTGATCAGTCACT-3′
primer16-f	5′-TTCTAAGGCCTGCCATGTCCA-3′
primer16-r	5′-TTTCCCCACTTAAGCAGAAGG-3′
primer17-f	5′-GAGGCTAGACGATGGAAATCA-3′
primer17-r	5′-TCCCAGATGCATGTATTTTCC-3′
primer18-f	5′-ACCAGTGACGTTTAAGTTGTT-3′
primer18-r	5′-AGAAAGCATGCGTTTTGTACT-3′

### mRNA microarray analysis

mRNA array analysis was performed in MDA-MB-231 cells transfected with either pcDNA3 vector carrying NBAT1 or an empty vector as negative control. Three pairs of independent samples were used for mRNA array analysis. After transfection for 72 h, total RNA was harvested using Trizol (Invitrogen) according to the manufacturer's instructions. Microarray expression profiles were collected using Agilent Whole Human Genome Microarray 4×44K.

### Data source

NBAT1 expression data were collected from public database of RNA sequencing data in The Cancer Genome Atlas (TCGA) pilot project which is established by the National Cancer Institute (NCI) and National Human Genome Research Institute (NHGRI) (https://tcga-data.nci.nih.gov/tcga/dataAccessMatrix.htm). A total of 716 breast cancer tissue and 98 normal tissues (breast cancer-normal tissue, BRCA-NT) were obtained from TCGA database. Expression data of NBAT1 was further extracted using LncRNAtor [12].

### Statistics

All statistical analyses were done using SPSS for Windows version 19.0. Student's *t*-test and two-way analysis of variance were used to correlate NBAT1 level with cancer metastasis, whereas χ^2^ test was applied to analyse the association between NBAT1 expression and clinicopathological status. Kaplan–Meier survival curves were plotted, and log rank test was done. All experiments in vitro were performed independently for at least three times and in triplicate for each time. All results are expressed as mean±s.d.. Student's *t*-test was used for the comparison of two independent groups. *P* value < 0.05 was considered statistically significant in all cases.

### Accession numbers

The Gene Expression Omnibus database accession number for the array data reported in this paper is GSE68034.
